# Effects of flow restoration on mussel growth in a Wild and Scenic North American River

**DOI:** 10.1186/2046-9063-9-6

**Published:** 2013-03-01

**Authors:** Brandon J Sansom, Daniel J Hornbach, Mark C Hove, Jason S Kilgore

**Affiliations:** 1 Oklahoma Biological Survey and Department of Biology, University of Oklahoma, 111 East Chesapeake St, Norman, OK, 73019, USA; 2 Department of Environmental Studies, Macalester College, St. Paul, MN, USA; 3 Department of Biology, Macalester College, St. Paul, MN, USA

**Keywords:** Unionidae, Annuli, Growth processing methods, Hydroelectric dam, Flow regulation

## Abstract

**Background:**

Freshwater mussels remain among the most imperiled species in North America due primarily to habitat loss or degradation. Understanding how mussels respond to habitat changes can improve conservation efforts. Mussels deposit rings in their shell in which age and growth information can be read, and thus used to evaluate how mussels respond to changes in habitat. However, discrepancies between methodological approaches to obtain life history information from growth rings has led to considerable uncertainty regarding the life history characteristics of many mussel species. In this study we compared two processing methods, internal and external ring examination, to obtain age and growth information of two populations of mussels in the St. Croix River, MN, and evaluated how mussel growth responded to changes in the operation of a hydroelectric dam.

**Results:**

External ring counts consistently underestimated internal ring counts by 4 years. Despite this difference, internal and external growth patterns were consistent. In 2000, the hydroelectric dam switched from operating on a peaking schedule to run-of-the-river/partial peaking. Growth patterns between an upstream and downstream site of the dam were similar both before and after the change in operation. At the downstream site, however, older mussels had higher growth rates after the change in operation than the same sized mussels collected before the change.

**Conclusions:**

Because growth patterns between internal and external processing methods were consistent, we suggest that external processing is an effective method to obtain growth information despite providing inaccurate age information. External processing is advantageous over internal processing due to its non-destructive nature. Applying this information to analyze the influence of the operation change in the hydroelectric dam, we suggest that changing to run-of-the-river/partial peaking operation has benefited the growth of older mussels below the dam.

## Background

Freshwater mussels are vital components to freshwater ecosystems and are considered ecosystem engineers because of their influence on surrounding habitat [1-3]. However, freshwater mussels are one of the most imperiled taxa in North America. Nearly 300 species are present in the United States, but 72% of these are endangered, threatened, or of special concern [4,5]. Much of this decline has been linked to habitat loss or degradation [6,7], a leading cause of which has been the impoundment of streams and rivers [7,8]. Numerous studies have documented the negative effect of dams on downstream mussel communities [9-12] with dozens of mussel species being extirpated [7]. Non-point source pollution and invasive species (e.g. zebra and quagga mussels) are additional factors known to negatively affect freshwater mussels [5].

Since changes in habitat impacts mussel life history traits knowing how mussels respond to these changes are crucial for proper management [13]. During periods of growth, mussels deposit calcium carbonate in the form of aragonite or calcite, resulting in shell construction [14]. The extent of calcium carbonate deposition is dependent on a number of environmental factors including water temperature [15-17], food availability and quality [3,18-21], and discharge [22]. Additionally, habitat features such as substrate type [23], stream length [16], water depth [24], riparian vegetation [25], and density of the surrounding mussel community [26,27] have also been known to influence variation in growth. Mussels grow in a similar manner to trees, in which rings are deposited when favorable growth conditions cease. During cessation of growth in mussels, an insoluble organic residue forms at the interface of the mantle and shell, leaving behind a conspicuous dark growth ring, which is observable on both the internal and external surfaces [14]. Proper examination and analysis of these rings in accordance with subsequent environmental conditions are useful in providing age and growth estimates.

While ring deposition has long been known in freshwater mussels [28], there is a lack of age and growth information for many mussel species, partly due to methodological issues associated with obtaining accurate age estimates and debate over the rate of ring deposition [29]. Recent work has focused on validating whether or not ring production occurs on an annual basis, a fundamental prerequisite to growth studies [30]. Various studies have examined this issue by using mark-recapture (see [31-33]) or dendrochronology applications [22,34-36]. Mark-recapture can be effective but is a time intensive method, and the possibility of low return of marked individuals may render results inconclusive [32,37]. More recent approaches utilize cross dating, a fundamental tool in dendrochronology. Cross dating uses high-frequency patterns in ring width variation to align each individual specimen’s series of growth increments to specific calendar years [38,39]. If growth within a population is synchronous, individual time series are averaged to create a master chronology (see [22,34-36]). Furthermore, false or missing ring(s) within a time series can be detected by comparing each individual to the master chronology. Finally, the growth rings of an individual mussel can be considered validated if its time series is significantly correlated to the master chronology [39]. A much less time-intensive method than mark-recapture, cross dating offers a unique way to validate ring deposition and has been successful for many species [22,35,36].

Traditional methods to view and interpret growth rings involve creating shell thin sections to view the internal growth rings. Viewing internal rings is useful for validating ring deposition rate with dendrochronology approaches, estimating age and growth rate, and identifying disturbance or false rings. Disturbance rings can be differentiated from true growth rings because annual growth rings are continuous from the shell exterior to the umbone and are broader and lighter in color than false rings [32]. However, generating thin sections requires destructive sampling, which is especially problematic for rare or endangered species. Furthermore, creating and examining thin sections is a tedious and time-consuming process.

Growth rings deposited by mussels are also prominent on the external surface of the shell and may provide an alternative method to interpret age and growth. External rings can be easily distinguished during the early years of growth but become crowded and difficult to differentiate towards the shell margin [32,33]. Additionally, disturbance rings can also appear on the external shell surface [17], and, although disturbance rings are usually thinner than true annuli, differentiating external disturbance from true annuli is difficult [32]. Furthermore, only a few studies have attempted to validate annual external ring production, and most do not observe annual production [17,32,40]. In fact, only one study, Ghent et al. [31], has conclusively demonstrated that external rings are formed annually.

Although external ring analysis may have limitations if only directly viewing and counting rings, cross dating potentially offers a valid approach to analyze general growth patterns in external rings. Because cross dating identifies frequency patterns of growth, a species of mussels with synchronous external growth patterns would suggest a regular rate of ring formation. Further work, such as confirmation through mark-recapture, would be needed to validate if external rings formed on an annual basis, but general growth information can be obtained by the periodicity of external ring formation. Therefore, if general growth patterns can be obtained from external rings, mussels will not have to be destructively sampled to analyze growth dynamics, an obvious benefit especially when working with threatened species.

In this study, we examined internal and external growth patterns of a common mussel species at two sites separated by a hydroelectric dam in the St. Croix River, MN. The first objective of the study was to examine the consistency of deriving age and growth rates between internal and external annuli for a Minnesota threatened mussel species, the mucket (*Actinonaias ligamentina*). We then used estimated growth rates from external annuli to evaluate the growth-effects of changing dam operations by comparing growth between sites upstream and downstream of the dam in 1994 (peaking) and 2010 (run-of-the-river/partial peaking) and by evaluating the long-term variation of growth within the downstream site.

## Results

### Growth pattern analysis

**Figure 1:**
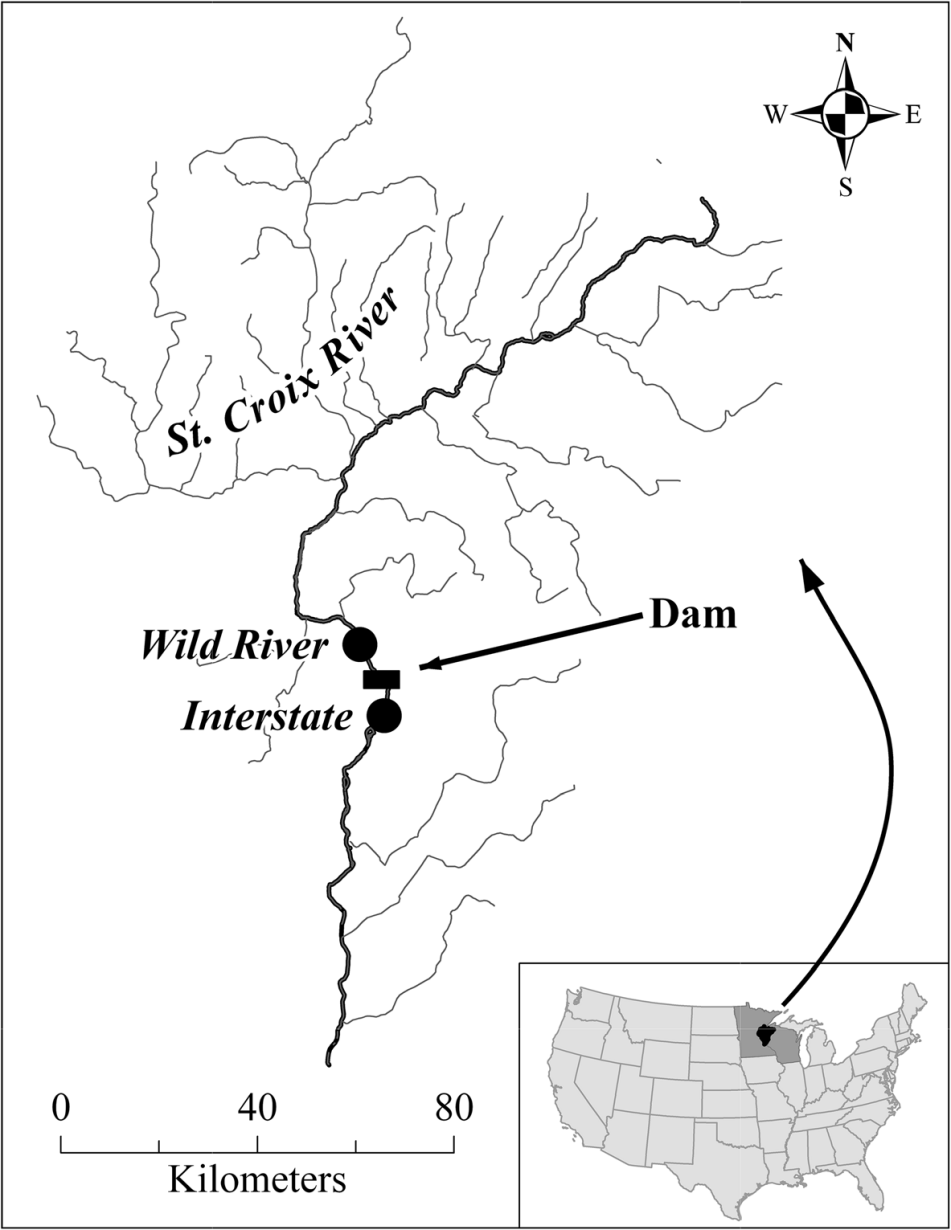
**Wild River and Interstate State Parks (Minnesota) study sites are located in the St. Croix River drainage basin.** The dam refers to the St. Croix Falls Hydro Generating Station.

**Figure 2:**
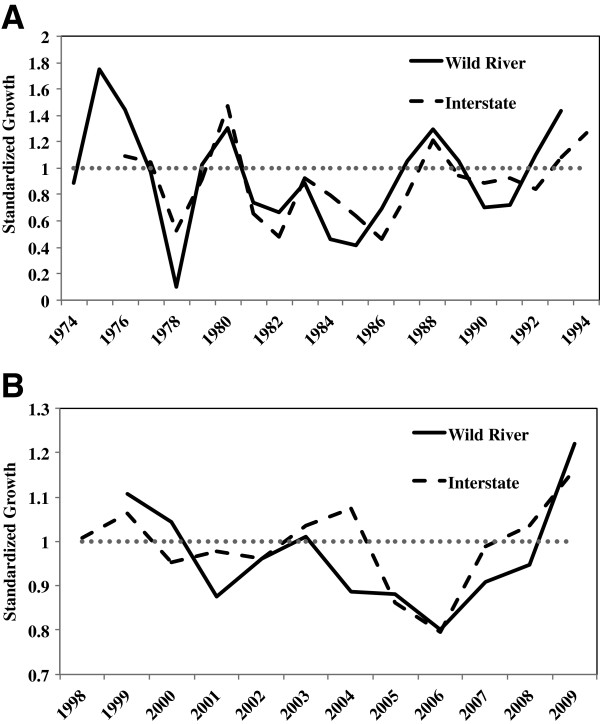
**Master chronologies depicting the yearly-standardized growth for (A) *Actinonaias ligamentina *collected in 1994 from Interstate State Park (*n *= 19) and in 1993 from Wild Rive State Park (*n *= 20) using internal processing, and (B) for *A. ligamentina *collected in 2010 depicting yearly-standardized growth from Wild River (*n *=11) and Interstate (*n *=12) State Parks using external processing methods.** Normal, expected growth is plotted on both representing the standardized growth index of 1. Values greater than 1 represent above average growth, whereas values less than 1 represent below average growth.

For all populations collected from Interstate State Park and Wild River State Park (hereafter, Interstate and Wild River, respectively; Figure 1), individual growth was highly synchronous for the entire population, resulting in strong interseries correlations indicative of a high degree of non-age-related growth variation among populations (Table 1). The internal ring master chronologies extended 19 years for Wild River (collected in 1993) and 18 years for Interstate (collected in 1994) (Figure 2A). The external ring master chronology extended 11 years for Wild River and 12 years for Interstate (both collected in 2010) (Figure 2B). Alternating periods of high and low growth were apparent in all populations, and for both collection dates (i.e. 1993/94 and 2010) there was a high degree of synchrony between the two locations (Figure 2A/B).

**Table 1:** **Cross dating statistics for *Actinonaias ligamentina *in the St. Croix River, MN**

					**Percent of individuals validated**		**Mean interseries *R *(Pearson’s)**	
**Location**	**Processing method**	**Time series**	**Optimal spline flexibility**	**n**	**Before quality control**	**After quality control**	**Before quality control**	**After quality control**
Interstate	External	1998-2009	34	33	42	42*	0.567	0.567**
	Internal	1975-1994	30	49	88	100	0.261	0.570
Wild river	External	1999-2009	24	35	43	43*	0.685	0.685**
	Internal	1974-1993	30	29	81	100	0.339	0.537

Note: *n*, the number of individuals validated for each population, that is all correlations are positive and significant; Optimal Spline Flexibility, the value resulting in the highest interseries correlation for each species; * Because mussels were returned to the river after processing, no specimens could be re-evaluated after using COFECHA; ** Only those individuals that initially were validated were used in the final data set for that population.

### Comparison of internal and external processing

**Figure 3:**
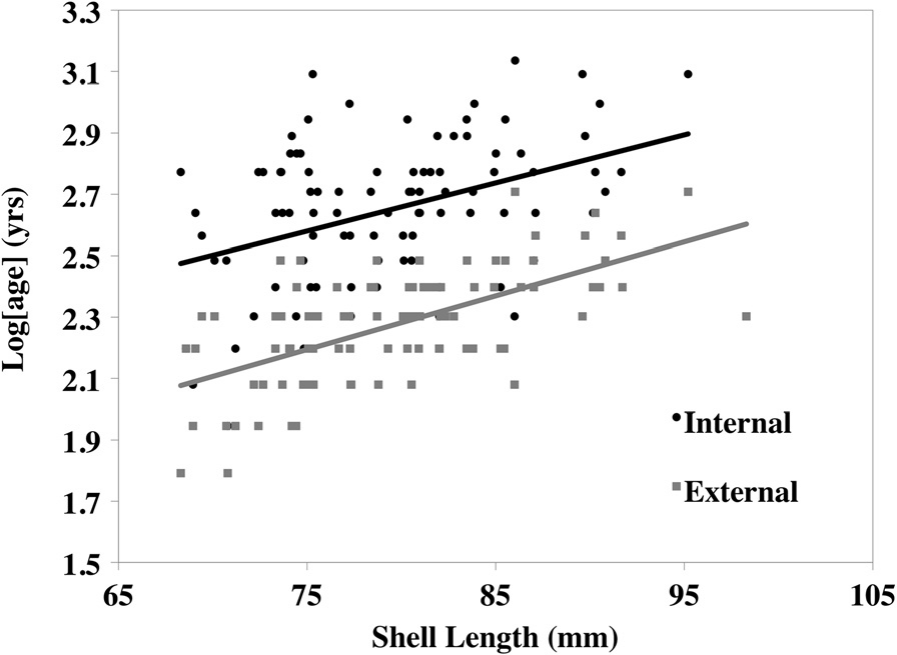
**Comparison of internal and external aging (natural log transformed) methods for *Actinonaias ligamentina *shells collected in 1994 from Interstate State Park.**

**Figure 4:**
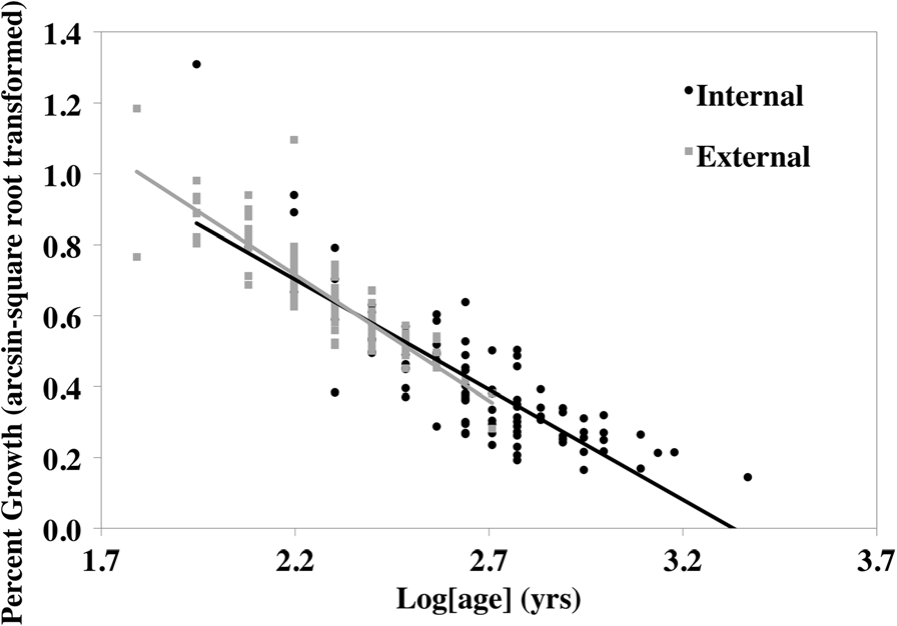
**Comparison of internal and external growth rates for *Actinonaias ligamentina *mussels collected in 1994 from Interstate State Park.** Age was natural log transformed.

Testing the consistency between internal and external ring processing methods on *A*. *ligamentina* collected from Interstate in 1994 yielded mixed results. Shell length and processing technique significantly influenced age estimation, but the interaction of length and technique was not significant (Figure 3; Length: *F* = 65.70, df = 1, 191, *p* < 0.0001; Age: *F* = 200.46, df = 1, 191, *p* < 0.0001). Relative to internal aging, external ring counts underestimated age by 4 years. Furthermore, because the interaction between length and technique was not significant, the difference in age was consistent for all sizes of mussels. Percent growth (arcsine-square root transformed) was not significantly influenced by technique or the interaction of technique and age (Figure 4). Age of mussel, however, did significantly influence percent growth (Figure 4; Log(age): *F* = 512.90, df = 1, 211, *p* < 0.0001).

### Influence of hydroelectric dam operation

Mean annual discharge was negatively correlated to internal growth at both Interstate and Wild River. This relationship was significant at Interstate (*R*^*2*^ = 0.32, *F* = 9.5, df = 18, *p* = 0.007). At Wild River, though not statistically significant, there was still a negative relationship (*R*^*2*^ = 0.14, *F* = 4.02, df = 19, *p* = 0.06). Water temperature data was only available from 2000 to 2010 and thus, only the growth chronologies obtained from the 2010 collection were correlated to growing degree-days. There was no significant relationship between growing degree-days and yearly growth for either Interstate or Wild River.

**Figure 5:**
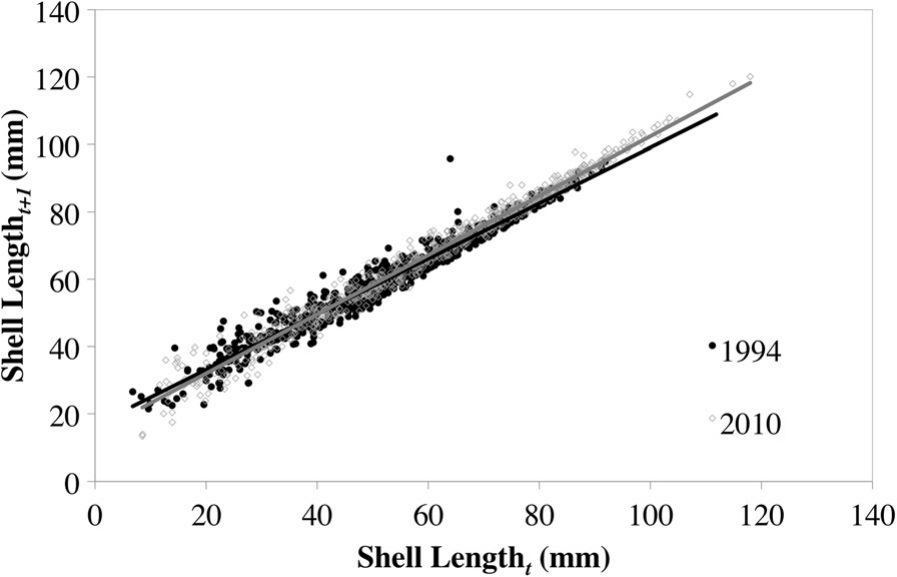
**A Ford-Walford plot comparing growth in 1994 and 2010 using external ring lengths of *Actinonaias ligamentina *mussels from Interstate State Park.**

Growth between Wild River and Interstate was similar for both collection dates (Figure 2A/B), as neither the location, year, nor their interaction had a significant influence on yearly growth. Despite similarities between Wild River and Interstate, there was a long-term variation in growth at Interstate (Figure 5). Using a Ford-Walford plot we found the length of the mussel, collection date, and their interaction had significant influences on growth (Year: *F* = 39.9, df = 1, 1539, *p* < 0.0001; Length_(*t*)_: *F* = 51079, df = 1, 1539, *p* <0.0001; Interaction: *F* = 54.8, df = 1, 1539, *p* < 0.0001). Growth was generally similar between collection dates for smaller mussels. However, larger mussels (>45 mm) collected in 2010 had higher growth than the same size of mussels collected in 1994. Furthermore, mussels collected in 2010 had a higher range of growth than mussels collected in 1994.

## Discussion

Our results suggest that useful growth information can be obtained from external growth rings of *Actinonaias ligamentina*. We found that percent growth was similar between different processing techniques, although age estimates were consistently different between internal and external aging methods. Using this information, we used growth patterns of external rings to examine the response of *A. ligamentina* to a change in dam operation from a peaking schedule to a run-of-the-river/partial peaking flow regime. The change to run-of-the-river/partial peaking appears to have benefited growth of older mussels below the dam.

The internal and external growth patterns obtained from this study meet the prerequisites of cross dating: *i*) growth shows non age-related patterns, and *ii*) individuals within a population exhibit synchronous growth [41]. Furthermore, identifying the regularity of ring deposition is essential to any growth study using organisms that form growth rings [30]. In cross dating studies for all taxa exhibiting ring deposition, a high degree of synchrony among individuals within a population indicates regular ring formation [22,39,41]. In this study, cross dating was an effective method to recognize synchronous growth patterns on both the internal and external surface of the shell. In addition, the strong interseries correlation for the master chronologies in this study suggests that the assumption of regular ring formation can be validated for both internal and external rings.

The rate at which rings form has important implications for management purposes. For instance, specific calendar years of growth could be aligned to corresponding climate data to determine how populations have responded to changes in the environment. Knowing if rings form annually further strengthens the ability to compare growth to specific environmental conditions. Ring formation in many mussel species, including *A. ligamentina*, is speculated to occur annually [32,42,43]. Using mark-recapture, Moles and Layzer [44] validated internal, annual ring formation of *A. ligamentina* in the Green River, KY. Moreover, cross dating has recently become an acceptable tool to validate annual internal ring formation for many mussel species [22,34-36]. Ideally, cross dating is used to verify annual ring production by either correlating growth to environmental variables or showing a correlation between two different chronologies of the same species, one in which annual ring formation has been validated using mark-capture and the other non-validated. Rypel et al. [22] and Black et al. [34] demonstrated a negative relationship between annual growth in freshwater mussels and mean annual discharge; thus mean annual flow is a reasonable variable to use to validate annual ring formation.

Mean annual discharge was negatively correlated to both internal chronologies from Interstate and Wild River in our study. Although the gauge used to obtain discharge is located below the dam (USGS gauge 05340500) and is not an ideal measurement for the population at Wild River, the strong negative relationship still suggests a pattern associated with internal ring formation. Furthermore, the interseries correlation for the internal chronology at Wild River was similar to that of the internal chronology at Interstate. Considering the negative correlation between mean annual discharge and both internal chronologies in this study, the prior validation of annual ring formation using mark-recapture in a separate population of *A. ligamentina*[44], and the consistency of interseries correlation among other species of mussels with validated annual ring formation [22,34-36] strongly suggests that the internal growth rings for *A. ligamentina* in this study are annual rings.

Whether or not external rings are produced annually cannot be determined from this study. There were no strong relationships between any of the external chronologies and mean annual discharge. Furthermore, no prior studies have validated external ring production using mark-recapture or cross dating studies with *A. ligamentina*. Only one mark-recapture study supports annual production of external rings; Ghent et al. [31] showed clear, conspicuous annual external ring formation for *Anodonta grandis*. Other studies, however, failed to examine external ring production to support this hypothesis [17,32,40]. Although we did observe high interseries correlation of our external chronologies, without having a direct relation to discharge or conclusive mark-recapture support, the significant interseries correlations can only confirm regular patterns of ring formation. Therefore, additional studies, ideally mark-recapture, would need to validate the rate of external ring formation with *A. ligamentina* in the St. Croix River.

This study supports recent studies [22,34,35] showing that cross dating is a powerful tool for identifying growth patterns within freshwater mussel populations. Although cross dating can be a lengthy and tedious process, it is still more efficient than traditional mark-recapture studies, and can provide larger sample sizes which yields stronger growth estimations for an entire population. Also, as with any living organism, mussels are susceptible to disturbance (e.g. flooding, predation, microhabitat changes, handling while processing) that can alter normal growth. When mussels experience such stress, growth may stop, and a disturbance ring is deposited [14]. Cross dating techniques can detect these false rings by comparing each individual series to the master chronology for that population, and thereby allowing for growth increments to be correctly aligned to specific calendar years.

The inconsistencies associated with age estimation between internal and external processing methods we found are similar to the findings of Neves and Moyer [33]. In our study, we estimated that external ages were consistently 4 years less than internal ages. Such differences in aging between processing techniques are likely due to difficulties in detecting external rings. In many of our specimens, the outer prismatic layer was eroded around the umbone, likely masking early juvenile growth rings. Additionally, older growth rings become crowded near the shell margin, making differentiation between subsequent rings difficult. Finally, external ring analysis occurs in a field setting likely introducing sampling error. Thus, failure of ring detection reduces the reliability for absolute age estimation using external rings.

Although internal aging is likely more accurate, we show that important growth information can still be obtained from external growth rings. To compare internal and external growth patterns, we first standardized growth measurements to account for differences in measurement techniques. External growth was measured along the anterior to posterior axis, while internal growth was measured dorsal to ventral, perpendicular to growth lines. Our methods of standardizing growth between internal and external processing allowed us to control for three factors influencing variation in growth. First, shell length and shell height were positively and significantly correlated (*R*^2^ = 0.73, *F* = 250, df = 95, *p* < 0.0001), suggesting that growth measurements were proportional across the shell. Because length and height are proportional, using these to standardize growth as a percent of size allowed us to control for measuring different axes. Second, by using the sum of the last 5 full years of consecutive, measurable growth, we were able to remove variation associated with age-related growth. This also helped control for the third factor, which was the difference associated with age between internal and external ring counts. Here, the increment between the rings, rather than the ring count, was important. Using the last 5 full years of growth, we were able to keep the same number of growth increments constant for both methods.

The consistent pattern of growth between internal and external processing methods (Figure 4) suggests two applications. First, despite a difference in ring counts, cross dating is still efficient at identifying patterns in growth for external rings. Because the rings that likely caused discrepancies in aging estimates were either at the beginning or end of an individual chronology, the difference in ring counts should not alter the ability to identify patterns in growth. This is supported by a high interseries correlation using external rings, which is also similar to both the chronology of the internal rings in this study and other studies using cross dating [22,34-36]. Our sampling methods did, however, limit our ability to conduct quality control with external processing. Once the external measurements were recorded, we immediately returned the mussels to the river. Doing so restricted us from re-examining specimens flagged for having potential false or missing rings on the external surface. Thus, we were only able to include those specimens that were initially positive and significantly correlated to the master chronology in order to reduce the possibility of including a false/missing ring. An easy remedy to improve quality control measures would be to mark mussels and keep them in an aerated tank until cross dating is complete in case any specimens need re-examined, then return the mussels to the river. Second, and perhaps most important, obtaining growth estimates from external rings offers a non-destructive sampling method.

Because of consistent growth patterns between internal and external processing, we were able to compare the impacts of the change in dam operation on the growth of mussels at Interstate using external ring data from 1994 and 2010. Numerous studies have documented negative effects on mussel communities downstream of dams [9,11,12]. Traditional dam operation, especially hydroelectric dams, is based on a peaking schedule that greatly disrupts the natural flow regime of rivers, resulting in disruptions of the species assemblage. Implementing a run-of-the-river operation schedule theoretically re-establishes a natural flow and should have positive impacts on mussel communities. Our results partially support this concept; there were similar growth patterns between both sites in 1994 and 2010 (Figure 2), but there was a long-term variation in growth below the dam before and after the implementation of a run-of-the-river/partial peaking schedule (Figure 5).

The similarities we found in growth patterns in both 1994 and 2010 between Wild River and Interstate are particularly interesting due to the presence of the hydroelectric dam between them. Recent mussel population surveys in the St. Croix River below the dam have documented declines of another common species (*Truncilla truncata*), but no declines in the *Actinonaias ligamentina* population have been observed [45]. This suggests that *A. ligamentina* are more tolerant to higher ranges of discharge or temperature. Another possibility for the similarities in growth between the Wild River and Interstate sites in 1994 could have been a result of mussels acclimating to the peaking schedule. The hydroelectric dam was installed in 1907, which would have given the mussels nearly a century to adjust to the altered flow regimes. There are a number of ways in which organisms are known to respond to changes in hydrological regime [46]. For example, life histories of the imperiled Pacific salmon vary depending on hydrologic regime [47]. While freshwater mussels are known to have changes in shell shape that respond to hydrologic regime [48], little is known regarding life history adaptations to changing flow. Though not statistically significant, growth did differ between Wild River and Interstate in 2010, especially during the first few years after the dam changed operation in 2000 (Figure 2B). This suggests that the mussels below the dam could be responding to the change in flow conditions due to run-of-the-river/partial peaking.

The long-term difference in growth (Figure 5) suggests that the change in dam operation has benefited the growth of larger mussels, while smaller mussels do not show much difference in growth between the two collection dates. Because we do not know the exact mechanisms controlling growth of *A. ligamentina* in the St. Croix River, we cannot infer why growth was higher for adults in 2010. It is possible that older mussels generally partition the majority of their energy resources to reproduction, but under more favorable conditions they have sufficient resources available for growth and reproduction. Layzer *et al*. [49] and Galbraith and Vaughn [9] both documented a reduction in fitness at flow-regulated study sites. In our study, streamflow conditions from the peaking schedule experienced by the mussels collected in 1994 could have resulted in lower food availability and higher fluctuations in water temperature and discharge [50]. In contrast, the natural flow regime mimicked by the run-of-the-river/partial peaking operations in 2010 could have provided a more consistent food supply and less variable, water temperature, and discharge, thus resulting in conditions more suitable for mussel reproduction and growth. Regardless of the precise mechanisms, our study indicates that the recent change in dam operation appears to benefit the growth of *A. ligamentina* in downstream reaches, particularly for older individuals.

## Conclusions

As habitat for freshwater mussels continues to be impaired, providing the best management strategies is crucial. Currently, knowledge of the predominant factors influencing mussel populations responses to changes in the environment are poorly understood. Annual rings deposited by freshwater mussels record temporal changes associated with their environment, and linking patterns in growth rings to environmental changes has important conservation implications. This study supports recent applications of cross dating as an effective tool for validating annual internal ring formation and obtaining more reliable estimates of mussel growth, at the individual or population level. Our study demonstrates that cross dating can be further applied to analyze patterns in external growth rings. Our methods of cross dating resulted in consistent growth patterns between internal and external processing methods. We suggest that similar cross dating methods can be applied to other mussel species, provided growth patterns within a population are highly synchronous. Cross dating also can be conducted using non-destructive sampling methods, which is especially beneficial for management and conservation activities related to threatened or endangered freshwater mussels species.

## Methods

### Study site

The St. Croix River, a National Wild and Scenic Riverway, drains approximately 20,000 km^2^ in east-central Minnesota and northwestern Wisconsin [51] and supports 41 species of freshwater mussels [52,53]. We collected *Actinonaias ligamentina* from Interstate and Wild River to reconstruct age and growth dynamics. *Actinonaias ligmanetina* is a common species throughout the United States with a life expectancy of around 50 years [44]. Interstate is located approximately 3.5 km downstream of a hydroelectric dam at St. Croix Falls, WI, while Wild River is about 10 km upstream the dam (Figure 1). The dam changed operation regimes from a partial hold-and-release schedule to run-of-the-river/partial peaking in 2000. The partial hold-and-released operated on a daily peaking schedule where water was stored and only released when sufficient amount was available to produce maximum energy output, whereas the run-of-the-river/partial peaking mimics a more natural, continuous flow, while still storing and releasing water for energy production. From 1910 to 2000, average discharge was 123 m^3^ s^-1^, with a range of 50 to 243 m^3^ s^-1^. Following operational changes, the average discharge was 126 m^3^ s^-1^ and ranged from 79 to 173 m^3^ s^-1^. Thus, although average discharge has remained relatively constant, the change to run-of-the-river/partial peaking has reduced the range of discharge considerably.

### Collection and measurement of shell rings

Mussels were collected from Wild River and Interstate in 1993 and 1994, respectively, as part of a physiological study. During that time, thin sections were prepared for the mussels from Wild River in contrast to only the shells being preserved from Interstate. These populations were re-sampled in 2010, but only external measurements were taken on live mussels and the mussels were returned to the river immediately after processing. Thus, methodology applied to measure shell rings (i.e. internal or external processing) was based on the availability of specimens, which varied among study sites and time periods: Wild River (1993: internal; 2010: external); Interstate (1994: internal and external; 2010: external), see also Table 1.

**Figure 6:**
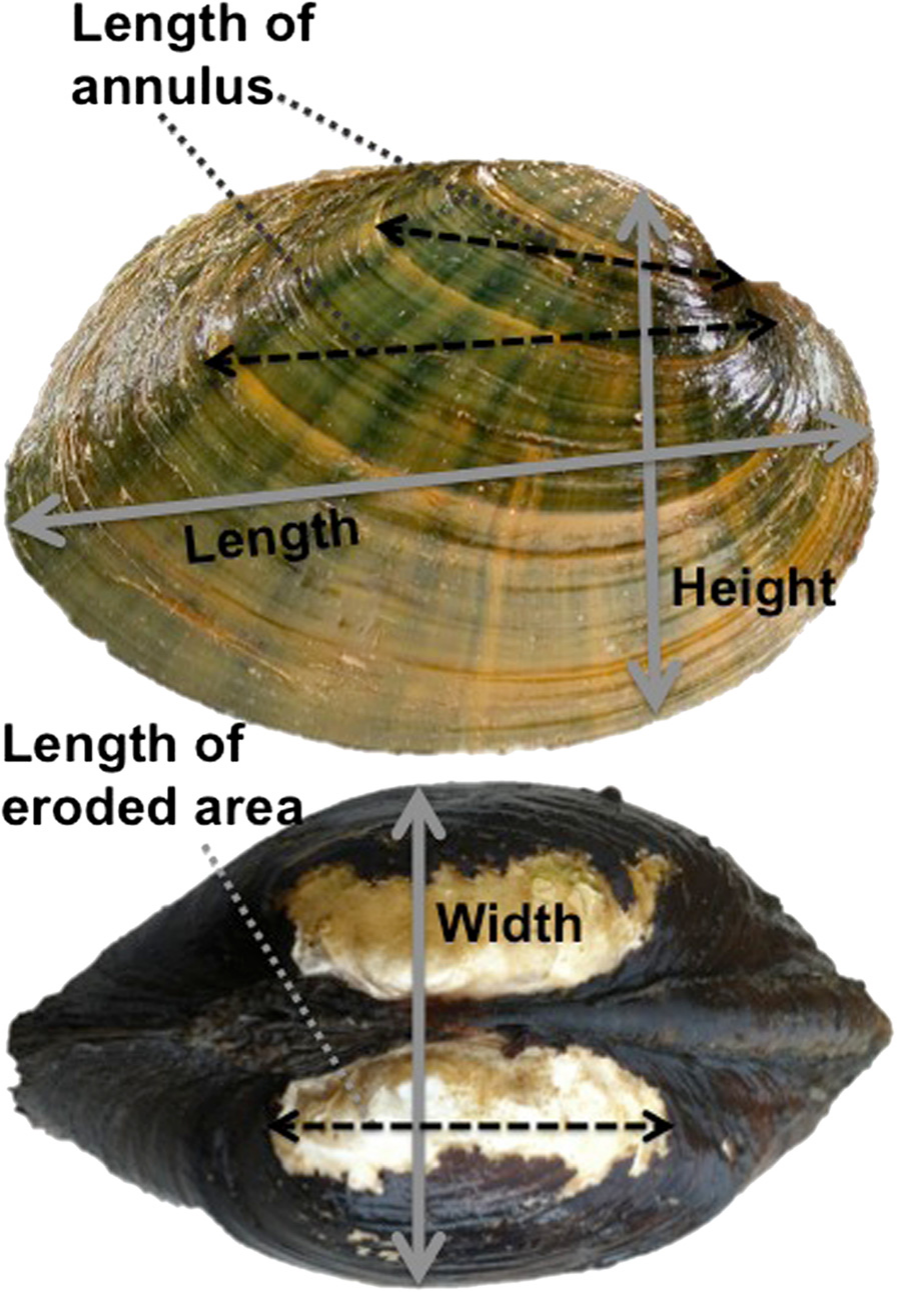
**The external processing measurements performed on *Actinonaias ligamentina*; length, height, and width of the total shell, the eroded area on the umbone, and the anterior to posterior length of each external annulus for each shell.**

External processing consisted of measuring the length, height, and width of the total shell, the eroded area on the umbone, and the anterior to posterior length of each external annulus for each shell (Figure 6). The same half of the shell (i.e. valve) that was used for external measurements was used for internal processing (applicable only to shells collected in 1994 from Interstate). Internal processing consisted of generating thin sections using standard methods for bivalves [33,54]. All thin sections were viewed and interpreted using a dissecting microscope (StemiDV4, Carl Zeiss, Gottingen, Germany). Internal annuli were identified, and the dorso-ventral growth increment along the prismatic and nacreous layers was measured using a linear encoder (ENC 150, Acu-Rite, Jamestown, NY) with a digital readout (QC-1000, Metronics, Bedford, NH). These measurements were linear in nature, not following the curve of the shell. Measurements began at the most recent complete growth year and continued towards the umbone. Disturbance (false) rings were identified as discontinuous from shell margin to the umbone and were not measured. Mussels < 5 years old were excluded from the sample because growth in early years is largely age-related and thus useful environmentally-influenced growth information is hard to obtain [35].

### Growth pattern analysis

Deposition rates of growth rings were validated using cross dating procedures following on Rypel *et al*. [22]. First, growth series for each individual were compared to the common signal (i.e. the average detrended value of shell ring increments for the population) to detect dating errors and to assess relative signal strength using COFECHA V1.26 (Dendrochronology Program Library; [39]). COFECHA removes age-related growth variation and generates a standardized index series by fitting a cubic spline to each series [39]. The optimum cubic spline depends on the average time length of the series. For each population, we determined the optimum cubic spline by choosing the value that yielded the highest interseries correlation for that specific population [22,41]; Table 1. From the standardized index, COFECHA generated a master chronology by taking the average growth index for each year. COFECHA then compares each individual time series to the master chronology, identifies potential problems with each series (e.g. false or missing annuli), and lags each time series both forward and backward to test if the series fits better at a different time interval. For internal growth chronologies, series flagged with potential problems were re-examined, and, if measurement errors were detected, the appropriate growth increments were re-measured [39]. After this quality control measure, COFECHA was then re-run with the corrected series(s). Quality control was limited for the external chronologies from 2010 due to sampling methods, as measurements were taken from live mussels that were immediately returned to the river once measurements were complete. Therefore, we were unable to re-examine any shells flagged as having errors by COFECHA. Consequently, only those individuals initially validated to the master chronology (Table 1) were used in our final data set. After quality control measures were complete, only those series that were both positively and significantly (α < 0.05) correlated to the master chronology were validated and included in the data set. The interseries correlation coefficient resulting from the validated individuals was used as a measurement of the strength of the common signal for the annual growth pattern for each population.

Using the raw, corrected growth measurements from COFECHA, data were transformed into standardized growth indices for each population using ARSTAN V1.26 (Dendrochronology Program Library; [55]). ARSTAN uses a detrending function to remove age-related growth and retain as much of the high-frequency variation in annual growth as possible. For this study, we used a negative exponential curve to detrend the growth measurements, resulting in a unitless, standardized growth index with an average value of 1 [22,55]. Growth values greater than 1 represented above average growth, whereas values less than 1 represented below average growth [56].

### Comparison of internal and external processing

Analyses of covariance (JMP statistical software, version 8.0, SAS Institute, Cary, NC) were used to evaluate the age and growth estimates derived from both internal and external processing methods for the *A. ligamentina* collected from Interstate in 1994. Age was estimated by counting the number of annuli on the external and internal surface, respectively. To remove age-related variation in the growth patterns, we developed a procedure to standardize raw growth measurements: the last 5 full, consecutive years of raw growth were summed and divided by total shell length or cross-section length, depending on internal or external measurements. External measurements were based on the anterior to posterior length of annuli; thus, for external measurements the total shell length was used to standardize growth. In contrast, internal measurements were measured perpendicular to the growth lines from the umbone to shell margin; thus, for internal measurements the total height (dorso-ventral dimension) was used to standardized growth.

### Influence of hydroelectric dam operation

We evaluated long-term patterns in growth downstream of the dam by comparing growth in 1994 to growth in 2010, as this could lead to further inferences about the impact of dam operation on mussel growth. We used a Ford-Walford plot [57], plotting length_(*t*)_ against length_(*t*+1)_, to model the growth of each population. An analysis of covariance (JMP statistical software, version 8.0, SAS Institute, Cary, NC) was used to compare growth rates between collection dates at Interstate. Because no internal measurements were taken in 2010, this analysis was performed only with external measurements. Finally, we obtained water temperature (converted to growing degree-days; Snyder, DegDay, University of California, version 1.01) and discharge data from USGS gauge 05340500 and used regression analysis (JMP statistical software, version 8.0, SAS Institute, Cary, NC) to compare growth to each.

## Competing interests

The authors claim no competing interests of this study.

## Authors’ contribution

BS participated in the collection and measurements of specimens, carried out the creation and measurement of thin section, and drafted the manuscript. DH coordinated the study, participated in the design of the study and collection and measurements of specimens, and helped draft the manuscript. MH participated in the study design, contributed to the collection and measurements of specimens, and made revisions to the manuscript. JK offered dendrological methods to improve analysis of growth patterns, participated in the interpretation and measurements of thin sections, and made revisions to the manuscript. All authors approved the final manuscript.
